# Clinical observation of specific changes of auricular points in patients with colorectal polyps: A case-control study

**DOI:** 10.1097/MD.0000000000044220

**Published:** 2025-09-05

**Authors:** Longshu Zhang, Ying Chen, Hui Li, Lirong Zhou, Mengyan Zhou, Xixia Zhang

**Affiliations:** a School of Nursing, Nanjing University of Chinese Medicine, Nanjing, Jiangsu Province, China; b Affiliated Hospital of Integrated Traditional Chinese and Western Medicine, Nanjing University of Chinese Medicine, Nanjing, Jiangsu Province, China; c Jiangsu Province Academy of Traditional Chinese Medicine, Nanjing, Jiangsu Province, China.

**Keywords:** auricular diagnosis, colorectal polyps, screening, specific changes of auricular points

## Abstract

To observe the specific changes of auricular points in patients with colorectal polyps (CPs) by auricular assessment. To summarize the clusters of auricular point-specific changes in patients with CPs, and to inform further research into auricular point assisted diagnosis of CPs. A total of 300 participants, with 150 having CPs and 150 having no CPs, were recruited for this case-control study. Auricular assessment included visual inspection, electrical skin resistance measurement, and tenderness testing. The chi-square test and binary logistic regression analysis were used to determine the association between categorical variables. And calculated the sensitivity, specificity, positive predictive value (PPV), and negative predictive value of each acupoint for CPs. Compared with the control group, the polyps group had more significant skin changes in the auricular point “Esophagus,” morphological changes in the auricular point “Stomach,” color changes in the auricular point “Duodenum,” color changes in the auricular point “Shenmen,” and shape changes in the auricular point “Rectum” observed on visual examination (*P* < .05). There was a significant difference in the electrical positive detection rate at 8 auricular acupoints, the “Large Intestine,” the “Appendix,” the “Small Intestine,” the “Kidney,” the “Pancreas and Gallbladder,” the “Center of Superior Concha,” and the “Shenmen” between the 2 groups (*P* < .05). In the polyps group, 4 auricular acupoints, the “Duodenum,” the “Large Intestine,” the “Small Intestine,” and the “Center of Superior Concha” showed more pronounced tenderness than in the control group. Among these auricular acupoints, the “Large Intestine” also showed the highest PPV of 61.5 on electrical skin resistance measurement, and the highest specificity of 53.3, PPV of 58.3, and negative predictive value of 60.6 on tenderness testing. This study showed that specificity changes in some of the auricular points such as the “Large Intestine,” the “Rectum,” the “Small Intestine,” the “Center of Superior Concha,” the “Shenmen,” and the “Kidney” are strongly correlated with the prevalence of CPs. Auricular diagnosis has a pre-diagnostic value. It is considered to establish the clusters of auricular point-specific changes to realize the early screening of CPs.

## 1. Introduction

### 1.1. Colorectal polyps

Intestinal polyps are elevated lesions on the surface of the intestinal mucosa that protrude into the intestinal lumen, which can occur in any part of the intestinal tract, most commonly in the rectum and the sigmoid colon.^[1]^ It can be divided into 4 categories according to the pathological type: adenomatous, misshapen tumor, inflammatory, and hyperplastic.^[2]^ Among them, adenomatous polyps account for 1/2 to 2/3 of all colorectal polyps (CPs), with a cancer rate of 9.4%,^[3]^ and are one of the common precancerous lesions of colorectal cancer. About 85% to 95% of colorectal cancers develop from adenomatous polyps.^[4]^ A Global Cancer Survey showed that there were more than 1.93 million new cases of colorectal cancer, which became the second leading cause of cancer deaths.^[5]^ The incidence of colorectal cancer in China has also shown a significant upward trend in recent years, and it has become the country with the highest number of new cases as well as the highest number of deaths from colorectal cancer in the world.^[6]^ Therefore, CPs, as one of the important precancerous lesions of colorectal cancer, deserve our high attention for screening and prevention.^[7]^

Colonoscopy is the gold standard for colorectal disease screening, which can reduce the incidence of colorectal cancer and its associated mortality by detecting it and performing endoscopic colorectal polypectomy.^[8]^

However, it is an invasive examination that requires complex intestinal preparations, such as taking intestinal cleansers like polyethylene glycol (PEG) electrolyte powder, and the following process of excretion is very painful. In some areas, the waiting period for appointments is relatively long (ranging from several weeks to several months), and the cost of a single examination is relatively high (painless colonoscopy costs between 1000 and 2500 RMB, and the charging standards vary among different regions and hospitals). This examination demands a high level of technical proficiency from the operating physician. In China, the number of medical institutions with corresponding hardware resources and qualified physicians is relatively small compared to developed countries. Coupled with patients’ fears and various emotional factors, a large proportion of the screening participants are reluctant to undergo colonoscopy.^[9]^ Therefore, we urgently need a more convenient, rapid and feasible screening method for CPs.

### 1.2. Auricular point therapy and auricular diagnosis

Auricular point therapy is a characteristic diagnosis and treatment technique of traditional Chinese medicine and an important branch of acupuncture, which has a long history in China.^[10]^ There is a record of “ear pulse” in “Yin-yang-11 Meridians Classic of Moxibustion,” which is the earliest known Chinese work on meridians. The famous Huangdi Neijing initially revealed the rudiments of theories related to auricular medicine, and applied auricular point diagnosis to disease diagnosis, prognosis, and guidance for the treatment.

Auricular diagnosis refers to when the internal organs or trunk of the human body have lesions, according to the corresponding region of the auricle appears a variety of pathological reactions, such as deformation, discoloration, desquamation, pimples, vascular changes, pressure and pain sensitivity and changes in the electrical properties of the skin (relevant auricular points of the skin electrical resistance value than the surrounding skin is reduced by about 10 to 50 times, so that the skin conductivity increases), etc to assist in the diagnosis of the disease.^[11]^ The claim that “A ear lobe crease (ELC) indicates coronary heart disease” was put forward by Frank,^[12]^ who also named the coexistence of cardiovascular risk factors and ELC the “Frank sign.” Subsequently, a large number of studies have also confirmed similar associations.^[13]^ Zhang Jin^[14]^ found in his clinical work that most patients with cholelithiasis would present with papules on the conchae of the ear. He used this as a criterion to diagnose cholelithiasis and found that the diagnostic coincidence rate reached 91.3%, successfully achieving the clinical transformation of auricular point diagnosis. These studies have clarified the value of auricular therapy in disease diagnosis.

Auricular acupoint therapy prevents and treats diseases by stimulating the acupoints on the auricle. Among the common auricular therapy methods for treatment are^[15]^: auricular bean pressing, auricular acupoint pressing, auricular acupuncture, electroacupuncture, bloodletting, laser, drug injection method, etc. A large number of studies have proved that its therapeutic effect is definite, such as auricular bean pressing for treating early knee osteoarthritis pain^[16]^ and gastrointestinal dysfunction,^[17]^ and auricular acupuncture for treating chronic low back pain^[18]^ and anxiety disorders.^[19]^

### 1.3. The application of auricular diagnosis in various diseases

Professor Lorna’s team^[13,20,21]^ had carried out a series of case-control studies on the correlation of auricular signals with coronary heart disease, lower urinary tract symptoms in men, and type 2 diabetes mellitus using a combination of auricular visual inspection, electrical skin resistance test and tenderness testing, confirming that the presence of an ELC was significantly associated with coronary heart disease.^[13]^ The pressure pain and electrical conductivity of some auricular points, including the “Pancreas and Gallbladder (P&G),” the “Endocrine” and the “Kidney” correlated significantly with type 2 diabetes mellitus status in Chinese population^[20]^; men with lower urinary tract symptoms tend to have pressure pain and decreased electrical conductivity of some auricular points, including the “Angle of Superior Concha,” the “Urinary Bladder,” the “Ureter”, and the “Internal Genitals.”^[21]^

The application of auricular point diagnosis in cancer patients has also achieved some results. Dr Lovato^[22]^ confirmed that auricular inspection is a simple and feasible method for breast cancer screening, and found that female breast cancer patients are more likely to develop auricular angioma in tumor area II. Zhu^[23]^ also found that patients with esophageal cancer may experience specific changes in some auricular points, which to a certain extent can also reflect the course of the cancer. As early as in the last century,^[24]^ a study was carried out in China on the changes of auricular current in patients with colorectal cancer and patients with intestinal polyps, and found that the auricular point of the “Large Intestine” and the “Colon” could be used as a reference for intestinal cancer and intestinal polyp census. A recent study of auricular electrodiagnosis in patients with CPs^[25]^ concluded that a positive correlation between the development of intestinal polyps and the electrometry results of auricular points the “Large Intestine,” the “Sanjiao,” the “Endocrine,” the “Center of Superior Concha,” and the “Liver.” The patients with CPs had a higher electrical positive detection rate in the “Large Intestine” and the “Sanjiao” than patients with non-CPs.

Based on this, it is necessary to apply auricular diagnosis, a convenient and noninvasive tool, to the early screening of CPs. There was a lack of relevant research on auricular point diagnosis for patients with CPs in the past. Therefore, a case-control study was conducted to examine the association of these auricular points-specific changes with CPs. Such a study would also facilitate extrapolation of the results to the colorectal cancer prevalent population and provide a basis for future studies examining the effectiveness of auricular diagnosis and treatment for colorectal cancer.

## 2. Aim and objectives

This study aims to investigate the association between the specific changes of 21 auricular points and the status of CPs among Chinese. The purpose of this study is as follows: to determine whether there are differences in vascular changes, skin changes, morphological changes and color changes on the surface of specific auricular points between patients with and without CPs; to confirm whether there are significant differences in the electrical positive detection rate on specific auricular points in patients with and without CPs; to identify whether there are significant differences exist on the tenderness in the specific auricular points between patients with and without CPs.

## 3. Methods

### 3.1. Settings and participants

This pilot study was a case-control study. The study recruited potential subjects from the Endoscopy Centre of Jiangsu Provincial Hospital of Integrated Traditional Chinese and Western Medicine (Nanjing, Jiangsu, China).

To calculate the sample size, we used the following comparing the means of 2 independent samples (α = 0.05, significance; β = 0.1; σ = 13.4; δ = 5.6) based on the available literature^[24]^ with maximum 20% falls during the follow-up, the final sample size was at least 150 participants per arm, 300 in total.


$$\begin{array}{l} \;\;\;n=\frac{{\left(Z_{\alpha }+Z_{\beta }\right)}^{2}*2\sigma^{2}}{\delta^{2}} \end{array}$$

The study adopted the convenient non-probability sampling method to select participants. From the start of the study, any patients who met the inclusion criteria and had no exclusion criteria were selected, and this process continued until the sample size was reached. A total of 405 people were recruited for the study. Those who did not meet the standards and had incomplete data collection were excluded. Finally, patients with colonoscopy findings of colorectal polyps (CPs+ve) were included in the observation group (n = 150), and those with colonoscopy showing no colorectal polyps (CPs−ve) were included in the control group (n = 150).

### 3.2. Selection criteria

The diagnostic criteria for CPs refer to the textbook “Surgery (9th Edition)” for 5-year undergraduate clinical medicine programs in Chinese higher education institutions.^[26]^ The intestinal polyps and intestinal polyposis are clinical diagnoses of a class of bulging lesions protruding from the surface of the mucosa into the lumen of the bowel, intestinal polyps can occur anywhere in the bowel, and CPs are most commonly found in the sigmoid colon and the rectum.

This study included those who had CPs (including CPs and multiple CPs) detected by colonoscopy or who underwent high-frequency electrocoagulation of intestinal polyps in the observation group, and those who did not have CPs by colonoscopy and did not suffer from any other major lower gastrointestinal diseases in the control group. All subjects were required to be in good general condition, free from serious diseases, aged 18 to 89 years, and of either sex.

The exclusion criteria were as follows: those who are unable to cooperate with auricular assessment; those who have inadequate bowel preparation with a Boston score of 9 or less thus affecting the endoscopist’s observation; those who suffer from other serious lower gastrointestinal diseases (including chronic colitis and ulcerative colitis, etc); those who have deformities and localized lesions in the ear (including scars, infections, ulcers, frostbite, breaks, etc) that affect the measurements; those who have a past and current history of mental illness or drug abuse.

### 3.3. Selection of specific auricular points

**Figure 1.**
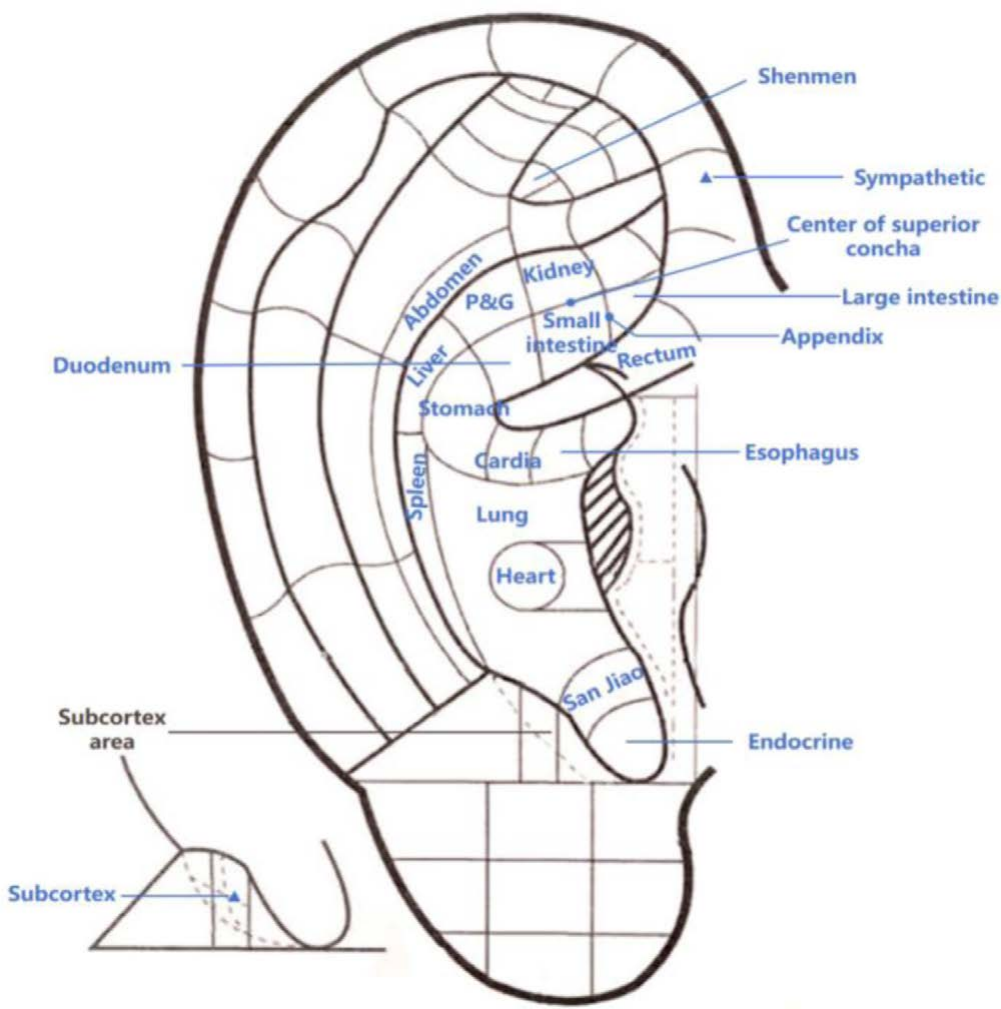
The 21 specific auricular points in the Chinese Standard Ear-Acupoint Chart. The picture is drawn based on the picture in “Nomenclature and location of auricular points: GB/ T13734-2008,” and the auricular points marked with names are those used in this study. P&G = Pancreas and Gallbladder.

Studies^[27]^ have found that the common clinical symptoms of patients with CPs include abdominal discomfort (abdominal pain, diarrhea, abdominal distension), changes in defecation habits and stool characteristics (hematochezia, bloody purulent stool), as well as general fatigue and anemia. Through screening the clinical studies on auricular point therapy for the treatment of above symptoms caused by digestive system diseases in the past 20 years, the auricular points in the studies that met the criteria of “Nomenclature and location of auricular points” (GB/T 13734-2008)^[28]^ and appeared more than 2 times were counted and sorted in descending order of frequency of appearance. Finally, the 21 auricular points associated with CPs were selected, including: “Large intestine,” “Stomach,” “Sympathetic,” “Shenmen,” “Spleen,” “Small intestine,” “San Jiao,” “Subcortex,” “Liver,” “Endocrine,” “Rectum,” “Kidney,” “P&G,” “Abdomen,” “Cardia,” “Heart,” “Duodenum,” “Lung,” “Esophagus,” “Center of superior concha,” “Appendix.” Figure 1 displays the location of these points.

### 3.4. Data collection

**Figure 2.**
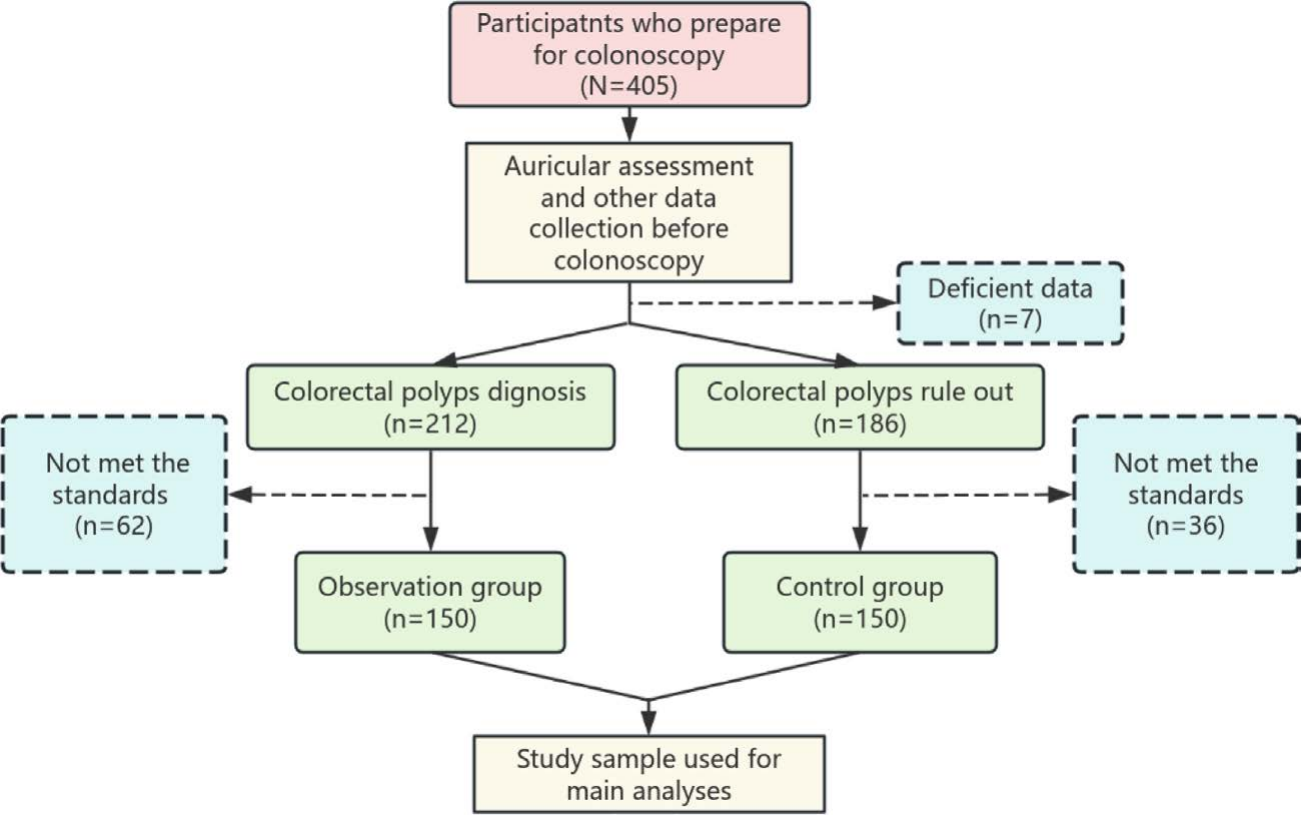
Flow chart of the study.

The basic information (age, gender, etc), medical history and family history of each participant were taken, and a health assessment was performed. The assessment covered the mental health status and intestinal preparation. After that, auricular assessment was performed in 3 ways: visual inspection, electrical skin resistance measurement (ESRM), and tenderness testing. The researcher involved in auricular examination was blinded to the grouping of subjects. A flow chart of the study design is presented in Figure 2.

#### 3.4.1. Instruction

(a) The mental health status of patients was evaluated using the 10-item Kessler Psychological Distress Scale (K10).^[29]^ Those with scores ranging from 10 to 19 were considered to have mild or no emotional distress and were suitable for inclusion in our study.
(b) The method of bowel preparation we adopted the method recommended in Consensus on bowel preparation for colonoscopy^[30]^: Taking 3 L of polyethylene glycol electrolyte (1 L at 8 pm the night before the test and 2 L 4 to 6 hours before the test) until clear watery stools are passed. If the stool consistency does not meet this requirement, additional doses can be taken, but the total amount generally should not exceed 4 L. When the patient made an appointment for a colonoscopy, we provided thorough education on the intestinal preparation plan and dietary restrictions, among other contents. Before the examination, we verbally asked the patient if they had passed clear watery stools and the time of their recent meals to assess their intestinal preparation.
(c) Data of the auricular assessment were collected by a traditional Chinese medicine practitioner with certified knowledge of auricular therapy (Chief TCM Physician) and the corresponding author of the study (who had received training in auricular point diagnosis and had decades of clinical experience, Chief Nurse). The auricular assessment was undertaken while adhering to the group standard “Technique specifications for treating weibing in Chinese medicine-Auricular point”^[31]^ formulated by the China Association of Chinese Medicine.
  - The process of visual inspection has been systematically organized by many experts and finally formed a widely recognized standard.^[31,32]^ The subjects and the researchers were under the irradiation of natural light. Did not clean, wipe, or knead the auricle before inspection. The examiner looked straight in the eyes, pinched the patient’s auricle with the thumb and index finger, and carefully examined whether there were the following 4 types of pathological reactions appearing on the selected auricular points^[33]^: Vascular changes such as bulging or interruption of blood vessels. Morphological changes such as nodules, folds, etc. Skin changes such as desquamation and pimples, etc. Color changes such as bright red, purplish-dark, and grayish-white, etc. In order to preserve the patient’s auricular point data, a high-resolution digital camera was used to obtain auricular photographs with the same shooting parameters during a relatively stable light period.
  - All subjects were operated on by the same researcher in a quiet state, under a relatively constant environment of temperature and humidity, and the ESRM was carried out after the subjects’ ears were wiped with 75% ethanol.^[31]^ The tool used for the ESRM was the patented instrument “SZF Auricular Point Detection Pen” (Manufacturer: Henan Shanzhifeng Information Science and Technology Co; Instrument Number: 20240625-4). This instrument employed an invention patent: a bioelectric health early warning system based on auricular points and its early warning method (CN116250820A), which has been applied in the collection and analysis of clinical auricular point data. Before the detection of selected auricular points, 5 nonselected points were selected for the determination of the basic value of skin resistance of individual ear acupuncture points, while the detection pen has the function of quantitatively displaying the impedance state of the points, and the electrical measurement of each point needed to wait for the electrical value to be stable for 1 to 2 seconds to ensure the accuracy of the resistance detection results. After all the points were tested, a test report was send to the connected electronic device, with negative results for resistance testing marked in green, weak positives in orange, and strong positives in red. The positive reaction points include weak positive and strong positive.^[25]^
  - Tenderness testing was conducted simultaneously with ESRM using the test pen. The probe of the pen was used to press the auricular points in sequence to assess the patient’s perception of pain at each point.^[34]^ Throughout the entire process, the probe pressure remained constant to ensure the same pressure, and the pressure pain was rated by the subject’s 4-point verbal rating scales.^[35]^ No tenderness was negative and rated as grade 0. Tenderness is a positive reaction, which can be divided into 3 grades according to the different degree of the nature of the pain: grade 1 for light pressure pain, that is, there was pain, but easily ignored; grade 2 is moderate pressure pain, that is, there is pain that cannot be ignored but can be tolerated; and grade 3 is severe pressure pain, that is, the pain is severe, and there is an autonomic avoidance or pushing away movement.

### 3.5. Validity and reliability

The researchers involved in data collection have conducted theoretical studies related to CPs and auricular diagnosis and treatment, and have received a more systematic course as well as hands-on learning of the testing equipment. The reliability of the auricular examination was assessed in 10 cases, and the researchers involved in data collection recorded the consistency of the observations, which was 95%. Where there were differences in assessment between the 2 raters, consensus was sought after discussion. The study was conducted in strict accordance with the study outline. A uniform guideline was used in the data collection phase to prevent information bias. During the auricular assessment, the data collection forms containing the subjects’ medical history were not accessible to the researchers to ensure blinding to subject grouping to avoid observer bias.

### 3.6. Data analyses

**Figure 3.**
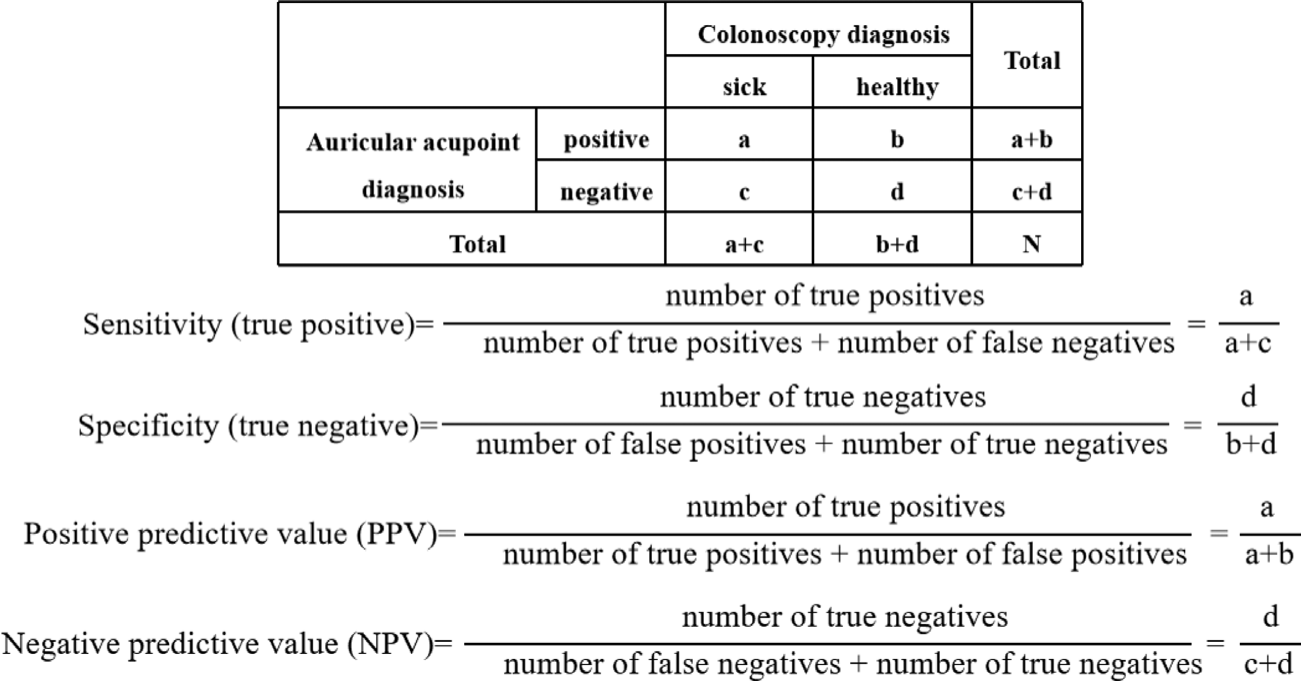
Calculation methods for sensitivity, specificity, PPV, and NPV. The drawing of the pictures was based on the calculation methods of sensitivity, specificity, PPV, and NPV in the references and the actual operation methods in this study. NPV = negative predictive value, PPV = positive predictive value.

The chi-square test was used to determine the association between auricular changes and colorectal polyps (+ve vs −ve). The 2 × 2 contingency tables were established to determine sensitivity, specificity, positive predictive value (PPV), and negative predictive value (NPV).^[36]^ The calculation method is shown in Figure 3. Binary logistic regression analysis was used to examine whether the incidence of CPs is associated with specific changes in auricular points. Analyses were conducted using SPSS version 27.0. A value of *P* < .05 was considered statistically significant.

### 3.7. Ethical considerations

The study received ethical approval from the Jiangsu Provincial Hospital of Integrated Traditional Chinese and Western Medicine, where the study was conducted. And it approved on the International Traditional Medicine Clinical Trial Registry (Registration number: ITMCTR2025000924). All participants signed written informed consent. The purpose and process of the study were explained to all subjects orally and in writing. Participation in the study was voluntary, and all participants were assured that they had the right to decline and withdraw from the study at any time. Personal information and data remained confidential and anonymous.

## 4. Results

### 4.1. Demographic characteristics of the participants

A total of 300 participants were recruited, among whom 150 were confirmed to have colorectal polyps (CP+ve) by colonoscopy and 150 were confirmed to have no colorectal polyps (CP−ve). The mean age of the participants was 52.07 years (SD = 11.29), with 151 males and 149 females.

There was no significant difference in gender and age between the 2 groups (*P* > .05), which was comparable (Table 1).

**Table 1:** Demographic characteristics of the participants.

	CP+ve (n = 150)	CP−ve (n = 150)	*P* value
Age	53.52 ± 10.85	50.61 ± 11.56	.366
Minimum	26	25	
Maximum	76	74	
Gender			
Male	79	72	.419
Female	71	78	

### 4.2. Association of specific acupoints and colorectal polyps status

Three methods of auricular point diagnosis were used to compare the specific changes of the auricular point between the case group (CP+ve) and the control group (CP−ve).

#### 4.2.1. Visual inspection

The specific changes of auricular points observed by visual inspection were classified into 4 categories: vascular changes, morphological changes, skin changes, and color changes. The difference between the CP+ve group and the CP−ve group subjects was statistically significant (*P* < .05) when comparing the skin changes of the “Esophagus,” the morphological changes of the “Stomach,” the color changes of the “Duodenum,” the color changes of the “Shenmen,” and the morphological changes of the “Rectum” (Table 2).

**Table 2:** Auricular changes between cases and controls assessed by visual inspection.

	CP+ve	CP−ve	*X* ^2^	*P* value
Esophagus Skin changes	15	5	5.357	.021*
	Desquamation (11) Acne (2) Bleeding (1) Hematoma (1)	Desquamation (4) Papule (1)		
Stomach Morphological changes	17	3	11.521	.001*
	Nodule (16) Pitting (1)	Nodule (3)		
Duodenum Color changes	12	3	5.684	.017*
	Light red (1) Bright red (5) Tawny (4) Purplish-dark (2)	Light red (1) Tawny (1) Purplish-dark (1)		
Shenmen Color changes	21	4	12.611	<.001*
	Light red (10) Bright red (8) Tawny (2) Purplish-dark (1)	Light red (3) Purplish-dark (1)		
Rectum Morphological changes	9	1	6.621	.024*
	Folding (9)	Folding (1)		

Fisher’s exact test was used if the expected values in any of the cells of a contingency table are below 5, or below 10 when there is only 1 degree of freedom.

* Statistically significant.

#### 4.2.2. Electrical skin resistance measurement

##### 4.2.2.1. Comparison of the electrical positive detection rate of right and left ear acupoints

To verify whether there was a difference in the electrical characteristics of auricular points in the left and right ears of all subjects, the subjects were randomly assigned in this study to collect the left ear acupoints by single-day visit and the right ear acupoints by even-day visit during data collection. A total of 147 cases were detected in the left ear and 153 cases in the right ear (Table 3). Generally, no significant differences were observed in the electrical positive detection rate of subjects’ 21 auricular points between the left and right ear (*P* > .05).

**Table 3:** Comparison of the electrical positive detection rate of right and left ear acupoints.

	Left (n = 147)		Right (n = 153)		*P*	*X* ^2^
	Negative (%)	Positive (%)	Negative (%)	Positive (%)		
Heart	58 (39.5)	89 (60.5)	62 (40.5)	91 (59.5)	.85	0.036
Lung	54 (36.7)	93 (63.3)	57 (37.3)	96 (62.7)	.926	0.009
Esophagus	47 (32.0)	100 (68.0)	36 (23.5)	117 (76.5)	.102	2.671
Cardia	39 (26.5)	108 (73.5)	45 (29.4)	108 (70.6)	.578	0.309
Stomach	63 (42.9)	84 (57.1)	69 (45.1)	84 (54.9)	.696	0.153
Spleen	76 (51.7)	71 (48.3)	94 (61.4)	59 (38.6)	.089	2.895
Endocrine	83 (56.5)	64 (43.5)	74 (48.4)	79 (51.6)	.160	1.97
Duodenum	83 (56.5)	64 (43.5)	80 (52.3)	73 (47.7)	.468	0.527
Large intestine	74 (50.5)	73 (49.7)	78 (51.0)	75 (49.0)	.912	0.012
Appendix	71 (48.3)	76 (51.7)	62 (40.5)	91 (59.5)	.175	1.837
Small intestine	53 (36.1)	94 (63.9)	67 (43.8)	86 (56.2)	.172	1.87
Kidney	56 (38.1)	91 (61.9)	58 (37.9)	95 (62.1)	.973	0.001
P&G	68 (46.3)	79 (53.7)	78 (51.0)	75 (49.0)	.413	0.669
Liver	83 (56.5)	64 (43.5)	83 (54.2)	70 (45.8)	.700	0.149
Center of superior concha	80 (54.4)	67 (45.6)	84 (54.9)	69 (45.1)	.933	0.007
Subcortex	83 (56.5)	64 (43.5)	76 (49.7)	77 (50.3)	.239	1.387
Shenmen	108 (73.5)	39 (26.5)	101 (66.0)	52 (34.0)	.160	1.972
Sympathetic	86 (58.5)	61 (41.5)	77 (50.3)	76 (49.7)	.155	2.02
Rectum	70 (47.6)	77 (52.4)	84 (54.9)	69 (45.1)	.207	1.592
San Jiao	75 (51.0)	72 (49.0)	74 (48.4)	79 (51.6)	.646	0.211
Abdomen	74 (50.5)	73 (49.7)	73 (47.7)	80 (52.3)	.649	0.207

Fisher’s exact test was used if the expected values in any of the cells of a contingency table are below 5, or below 10 when there is only 1 degree of freedom.

P&G = Pancreas and Gallbladder.

##### 4.2.2.2. Comparison of the electrical positive detection rate of the CP+ve group and the CP−ve group

Among the 21 electrical points tested, the electrical positive detection rate of 8 points, the “Large Intestine,” the “Appendix,” the “Small Intestine,” the “Kidney,” the “P&G,” the “Center of superior concha,” the “Subcortex” and the “Shenmen,” were significantly different between the CP+ve group and the CP−ve group (*P* < .05). In addition, patients in the CP+ve group showed a higher proportion of the electrical positive detection rate than the CP−ve group (Table 4).

**Table 4:** Auricular changes between cases and controls assessed by ESRM.

	CP+ve (n = 150)		CP−ve (n = 150)		*P*	*X* ^2^
	Negative (%)	Positive (%)	Negative (%)	Positive (%)		
Heart	63 (42.0)	87 (58.0)	57 (38.0)	93 (62.0)	.48	0.5
Lung	49 (32.7)	101 (67.3)	62 (41.3)	88 (58.7)	.12	2.417
Esophagus	38 (25.3)	112 (74.7)	45 (30.0)	105 (70.0)	.366	0.816
Cardia	43 (28.7)	107 (71.3)	41 (27.3)	109 (72.7)	.797	0.066
Stomach	60 (40.0)	90 (60.0)	72 (48.0)	78 (52.0)	.163	1.948
Spleen	86 (57.3)	64 (42.7)	84 (56.0)	66 (44.0)	.816	0.054
Endocrine	84 (56.0)	66 (44.0)	73 (48.7)	77 (51.3)	.204	1.617
Duodenum	74 (49.3)	76 (50.7)	89 (59.3)	61 (40.7)	.082	3.023
Large intestine	59 (39.3)	91 (60.7)	93 (62.0)	57 (38.0)	<.001*	15.416
Appendix	55 (36.7)	95 (63.3)	78 (52.0)	72 (48.0)	.008*	7.145
Small intestine	47 (31.3)	103 (68.7)	73 (48.7)	77 (51.3)	.002*	9.389
Kidney	43 (28.7)	107 (71.3)	71 (47.3)	79 (52.7)	<.001*	11.092
P&G	62 (41.3)	88 (58.7)	84 (56.0)	66 (44.0)	.011*	6.458
Liver	75 (50.0)	75 (50.0)	91 (60.7)	59 (39.3)	.063	3.453
Center of superior concha	67 (44.7)	83 (55.3)	97 (64.7)	53 (35.3)	<.001*	12.105
Subcortex	65 (43.3)	85 (56.7)	94 (62.7)	56 (37.3)	<.001*	11.254
Shenmen	95 (63.3)	55 (36.7)	114 (76.0)	36 (24.0)	.017*	5.694
Sympathetic	74 (49.3)	76 (50.7)	89 (59.3)	61 (40.7)	.082	3.023
Rectum	72 (48.0)	78 (52.0)	82 (54.7)	68 (45.3)	.248	1.334
San Jiao	69 (46.0)	81 (54.0)	80 (53.3)	70 (46.7)	.204	1.613
Abdomen	76 (50.7)	74 (49.3)	71 (47.3)	79 (52.7)	.564	0.333

Fisher’s exact test was used if the expected values in any of the cells of a contingency table are below 5, or below 10 when there is only 1 degree of freedom.

ESRM = electrical skin resistance measurement, P&G = Pancreas and Gallbladder.

* Statistically significant.

#### 4.2.3. Tenderness testing

The tenderness rating was divided into 4 grades, and it was found that the tenderness of these 4 auricular points, the “Duodenum,” the “Large Intestine,” the “Small Intestine” and the “Center of superior concha” had significant differences between the CP+ve group and the CP−ve group (*P* < .05), which showed that the CP+ve group had obvious tenderness at these auricular points (Table 5).

**Table 5:** Auricular changes between cases and controls assessed by tenderness testing.

	Group (n = 150)	Grade 0 (%)	Grade 1 (%)	Grade 2 (%)	Grade 3 (%)	*P*	*X* ^2^
Heart	CP+ve	65 (43.3)	63 (42.0)	20 (13.3)	2 (1.3)	.139	5.475
	CP−ve	76 (50.7)	44 (29.3)	27 (18.0)	3 (2.0)		
Lung	CP+ve	61 (40.7)	55 (36.7)	31 (20.7)	3 (2.0)	.451	2.635
	CP−ve	74 (49.3)	46 (30.7)	26 (17.3)	4 (2.7)		
Esophagus	CP+ve	67 (44.7)	48 (32.0)	33 (22.0)	2 (1.3)	.129	4.888
	CP−ve	82 (54.7)	43 (28.7)	25 (16.7)	0 (0.0)		
Cardia	CP+ve	72 (48.0)	45 (30.0)	32 (21.3)	1 (0.7)	.765	1.575
	CP−ve	79 (52.7)	41 (27.3)	30 (20.0)	0 (0.0)		
Stomach	CP+ve	75 (50.0)	47 (31.3)	26 (17.3)	2 (1.3)	.363	3.178
	CP−ve	78 (52.0)	36 (24.0)	35 (23.3)	1 (0.7)		
Spleen	CP+ve	84 (56.0)	44 (29.3)	22 (14.7)	0 (0.0)	.531	1.265
	CP−ve	77 (51.3)	44 (29.3)	29 (19.3)	0 (0.0)		
Endocrine	CP+ve	52 (34.7)	47 (31.3)	46 (30.7)	5 (3.3)	.052	7.472
	CP−ve	70 (46.7)	46 (30.7)	33 (22.0)	1 (0.7)		
Duodenum	CP+ve	49 (32.7)	60 (40.0)	40 (26.7)	1 (0.7)	.045*	8.005
	CP−ve	73 (48.7)	45 (30.0)	31 (20.7)	1 (0.7)		
Large intestine	CP+ve	52 (34.7)	49 (32.7)	47 (31.3)	2 (1.3)	.004*	12.358
	CP−ve	80 (53.3)	39 (26.0)	31 (20.7)	0 (0.0)		
Appendix	CP+ve	54 (36.0)	45 (30.0)	48 (32.0)	3 (2.0)	.084	6.625
	CP−ve	75 (50.0)	39 (26.0)	33 (22.0)	3 (2.0)		
Small intestine	CP+ve	53 (35.3)	44 (29.3)	50 (33.3)	3 (2.0)	.029*	8.917
	CP−ve	77 (51.3)	38 (25.3)	34 (22.7)	1 (0.7)		
Kidney	CP+ve	81 (54.0)	45 (30.0)	22 (14.7)	2 (1.3)	.193	3.956
	CP−ve	94 (62.7)	38 (25.3)	18 (12.0)	0 (0.0)		
P&G	CP+ve	87 (58.0)	38 (25.3)	25 (16.7)	0 (0.0)	.639	0.895
	CP−ve	95 (63.3)	33 (22.0)	22 (14.7)	0 (0.0)		
Liver	CP+ve	87 (58.0)	39 (26.0)	23 (15.3)	1 (0.7)	.946	1.139
	CP−ve	86 (57.3)	42 (28.0)	22 (14.7)	0 (0.0)		
Center of superior concha	CP+ve	52 (34.7)	36 (24.0)	56 (37.3)	6 (4.0)	.044*	8.039
	CP−ve	67 (44.7)	44 (29.3)	36 (24.0)	3 (2.0)		
Subcortex	CP+ve	53 (35.3)	38 (25.3)	53 (35.3)	6 (4.0)	.097	6.272
	CP−ve	63 (42.0)	48 (32.0)	36 (24.0)	3 (2.0)		
Shenmen	CP+ve	85 (56.7)	33 (22.0)	29 (19.3)	3 (2.0)	.836	0.854
	CP−ve	88 (58.7)	36 (24.0)	24 (16.0)	2 (1.3)		
Sympathetic	CP+ve	85 (56.7)	37 (24.7)	27 (18.0)	1 (0.7)	.751	1.580
	CP−ve	92 (61.3)	34 (22.7)	24 (16.0)	0 (0.0)		
Rectum	CP+ve	60 (40.0)	49 (32.7)	35 (23.3)	6 (4.0)	.538	2.164
	CP−ve	72 (48.0)	42 (28.0)	32 (21.3)	4 (2.7)		
San Jiao	CP+ve	74 (49.3)	47 (31.3)	26 (17.3)	3 (2.0)	.224	4.315
	CP−ve	88 (58.7)	34 (22.7)	27 (18.0)	1 (0.7)		
Abdomen	CP+ve	92 (61.3)	36 (24.0)	22 (14.7)	0 (0.0)	.567	1.135
	CP−ve	95 (63.3)	29 (19.3)	26 (17.3)	0 (0.0)		

Fisher’s exact test was used if the expected values in any of the cells of a contingency table are below 5, or below 10 when there is only 1 degree of freedom.

P&G = Pancreas and Gallbladder.

* Statistically significant.

### 4.3. Predictive power of various acupoints for colorectal polyps

The predictive power of individual auricular points for CPs was calculated for each of the 3 measurements that were significantly different between the CP+ve group and the CP−ve group (Table 6).

**Table 6:** Predictive power of various acupoints for colorectal polyps by 3 methods.

	Sensitivity (%)	Specificity (%)	Positive predictive value (%)	Negative predictive value (%)
Visual inspection				
Esophagus Skin changes	10.0	96.7	75.0	51.8
Stomach Morphological changes	12.0	98.0	85.7	52.7
Duodenum Color changes	8.0	98.0	80.0	51.6
Shenmen Color changes	**14.0**	97.3	84.0	**53.1**
Rectum Morphological changes	6.0	**99.3**	**90.0**	51.4
Electrical skin resistance measurement				
Large intestine	60.7	62.0	**61.5**	61.2
Appendix	63.3	52.0	56.9	58.6
Small intestine	68.7	48.7	57.2	60.8
Kidney	**71.3**	47.3	57.5	**62.3**
P&G	58.7	56.0	57.1	57.5
Center of superior concha	55.3	64.7	61.0	59.1
Subcortex	56.7	62.7	60.3	59.1
Shenmen	36.7	**76.0**	60.4	54.5
Tenderness testing				
Duodenum	**67.3**	48.7	56.7	59.8
Large intestine	65.3	**53.3**	**58.3**	**60.6**
Small intestine	64.7	51.3	57.1	59.2
Center of superior concha	65.3	44.7	54.1	56.3

Figures in bold indicate the highest among the acupoints under testing.

P&G = Pancreas and Gallbladder.

#### 4.3.1. Visual inspection

The color changes of the “Shenmen” exhibited the highest sensitivity of 14.0% and an NPV of 53.1%. At the same time, the morphological changes of the “Rectum” had the highest specificity of 99.3% and a PPV of 90.0%.

#### 4.3.2. Electrical skin resistance measurement

The electrical positive detection rate of the “Kidney” showed the highest sensitivity of 71.3% and NPV of 62.3%, and the “Shenmen” showed the highest specificity of 76.0%. The “Large Intestine” had the highest PPV of 61.5%, and the PPV of the “Center of superior concha” was slightly lower than the “Large Intestine” (61.0%).

#### 4.3.3. Tenderness testing

Besides the “Duodenum” had the highest sensitivity of 67.3%, the “‘Small intestine’” demonstrated considerable specificity (53.3%), PPV (68.3%), and NPV (60.6%) during the tenderness testing.

### 4.4. Multivariate analysis

The diagnosis of CPs was taken as the dependent variable, and the statistically significant specific changes of auricular point in the above results were taken as independent variables for multivariate logistic regression analysis (Table 7). The results showed that the skin changes of the “Esophagus” (OR = 3.391), the morphological changes of the “Stomach” (OR = 7.051), the color changes of the “Duodenum” (OR = 4.917), the color changes of the “Shenmen” (OR = 4.742), the morphological changes of the “Rectum” (OR = 13.883), the positive electrical detection of the “Large Intestine” (OR = 2.243) and the tenderness of the “Small intestine” (OR = 1.434) were independently correlated with CPs.

**Table 7:** Multivariate logistic regression analysis of colorectal polyps diagnosed by 3 methods.

	Odds ratio	95% CI	*P* value
Visual inspection(Absent = 0, Present = 1)			
Esophagus Skin changes	3.391	1.005–11.447	.049*
Stomach Morphological changes	7.051	1.769–28.099	.006*
Duodenum Color changes	4.917	1.177–20.541	.029*
Shenmen Color changes	4.742	1.42–15.837	.011*
Rectum Morphological changes	13.883	1.389–138.768	.025*
Electrical skin resistance measurement (Negative = 0, Positive = 1)			
Large intestine	2.243	1.156–4.354	.017*
Appendix	0.842	0.379–1.872	.673
Small intestine	1.326	0.67–2.62	.418
Kidney	1.42	0.661–3.05	.368
P&G	0.853	0.437–1.664	.641
Center of superior concha	1.162	0.599–2.254	.657
Subcortex	1.588	0.825–3.057	.166
Shenmen	1.05	0.533–2.067	.888
Tenderness testing (Negative = 0, Positive = 1)			
Duodenum	1.183	0.842–1.663	.332
Large intestine	1.374	0.982–1.924	.064
Small intestine	1.434	1.035–1.985	.03*
Center of superior concha	1.154	0.853–1.561	.353

CI = confidence interval, P&G =Pancreas and Gallbladder.

* Statistically significant.

## 5. Discussion

In this study, we comprehensively used the methods of visual inspection, ESRM, and tenderness testing on 21 auricular points to explore the correlation between specific changes of auricular points and CPs.

### 5.1. The theoretical basis of auricular points

Traditional Chinese medicine believes that the ear is closely related to the meridians and viscera of the whole body. The *Huangdi Neijing* recorded: “ The ear is where many meridians converge.” The ear is an important external phase of the viscera of the human body. The essence of the auricular point is the information point corresponding to each organ and tissue of the human body on the auricle.

It was Dr Paul NOGIER from France who systematized auricular point therapy. The first step in the establishment of his theoretical system was the formulation of the doctrine of the “inverted embryo” of the auricle. He believed that each response point in the ear corresponded to a specific area of the body, and the distribution of reflection areas is similar to the shape of an inverted baby.^[32]^ Later, to promote cultural exchanges between China and foreign countries and to facilitate research by scholars, the China Acupuncture Standardization Technical Committee unified the positioning and function of auricular points, and the China Standardization Organizing Committee issued the latest updated “Nomenclature and location of auricular points”^[28]^ in 2008. In that, we can also see that the distribution of the ear points is in the shape of an inverted body.

The anatomical structure of the external ear mainly consists of 8 parts: the helix, the tragus, the conchae, the anthelix, the antitragus, the fossa anthelicis, the scapha, and the lobulus auriculae. Among them, a large number of auricular points related to the digestive system are distributed in the ear conchae area, such as the “Mouth,” the “Esophagus,” the “Cardia,” the “Duodenum,” the “Small Intestine,” the “Large Intestine,” the “P&G,” the “Liver,” the “Spleen” and so on. At the same time, the ear conchae area is also the only superficial location where the auricular branches of the vagus nerve are distributed in the human body,^[37]^ which is generally considered to be the best location for body surface stimulation. The vagus nerve is the longest pair of cerebral nerves in the human body, which is in charge of the human body’s digestive and respiratory systems, as well as the related glands. So it plays a role in regulating gastrointestinal nerves. At present, vagus nerve stimulation and a new type of ear acupuncture therapy-transcutaneous auricular vagus nerve stimulation, have been proven to be effective in alleviating symptoms of functional gastrointestinal disease and preventing postoperative intestinal obstruction.^[38]^ These studies have confirmed that the auricular concha area is closely related to human digestive system diseases. The above can be used as the theoretical basis for the auricular point reaction of intestinal diseases.

### 5.2. Visual inspection

The visual examination of auricular points begins with an overall observation of the auricle, that is, the color and morphological changes of the outer ear. Then, the inspection of specific areas on the surface of the auricle, namely auricular points, was carried out. The changes mainly focused on deformation, discoloration, desquamation, papules, and vascular filling.^[33]^ Compared with the control group, the skin changes of the “Esophagus,” the morphological changes of the “Stomach,” the color changes of the “Duodenum,” the color changes of the “Shenmen,” and the morphological changes of the “Rectum” were obvious in patients with CPs. Among them, the color changes of the “Shenmen” (mainly bright red) and the morphological changes of the “Rectum” (mainly folds) were more significant in predicting CPs. In years of clinical diagnosis experience, it has been found that discoloration reaction accounts for about 40% of visual inspection positive reactions, and the bright red change usually represents acute and painful diseases. The “Shenmen” has obvious analgesic, sedative, and anti-inflammatory effects,^[39]^ and inflammatory polyps are a common pathological type in CPs. At the same time, the bright red color change of the “Shenmen” can be argued for the abdominal pain, which is a relatively obvious symptom in the early stage of this disease.

Rectum is one of the initial disease sites of CPs, and the morphological changes of the “Rectum” once again proved that it has the function of reflecting intestinal diseases. The “Rectum” usually has the effect of clearing heat and dampness, as well as regulating viscera and astringenting the intestine. It is often used in clinical practice to treat diarrhea or constipation and other diseases. However, some scholars believe that the appearance of folds is related to surgical history.^[40]^ Because CPs have the characteristic of being prone to recurrence, some of the subjects in this study did have a history of CP resection. As for whether the folds on the “Rectum” are related to their history of polyp removal, it cannot be asserted at present. Following the discovery by Frank^[12]^ that there is a certain correlation between the ELC and coronary heart disease, many studies have been published supporting the view that ELC is independently related to the prevalence and severity of coronary heart disease.^[13,41]^ However, as a supplementary point raised in these studies, the appearance of folds may be related to ear lobe shape, age, and race,^[42]^ so this issue also had to be considered in this study. The study was conducted only in China, so the results may be difficult to apply to other ethnic groups. At the same time, the oldest age of patients with CPs in this study has reached 78 years, and the average age has also reached 53 years. The appearance of the folds on the “Rectum” is also very likely to be related to skin relaxation caused by age.

### 5.3. Electrical skin resistance measurement

Compared with the control group, the electrical positive detection rate of CPs patients were higher in the following auricular points, the “Large Intestine,” the “Appendix,” the “Small Intestine,” the “Kidney,” the “P&G,” the “Center of superior concha,” the “Subcortex” and the “Shenmen.” That is, the resistance of these auricular points becomes lower. Among them, the prediction ability of the “Kidney” for CPs was significant, while the positive prediction value of the “Large Intestine” and the “Center of superior concha” was higher. This result was also consistent with the research results of Du et al,^[25]^ which confirmed that the large intestine is one of the initial disease sites of CPs. It shows that the changes in electrical characteristics of the “Large Intestine” could indeed reflect the disease status of CPs. The “Center of superior concha” is an important reference auricular point for the diagnosis of abdominal diseases. Clinically, it has been found that people with long-term constipation, diarrhea, or repeated alternating occurrences are more likely to have chronic inflammation of the intestinal mucosa and be more prone to polyps.^[25]^ This point is exactly consistent with the function of this auricular point in treating abdominal pain, abdominal distension, and diarrhea. The “Kidney” is usually used in clinical treatment of kidney disease or endocrine system disease, and has the effect of “Regulating qi and removing dampness.” At the same time, both the “Large Intestine,” and the “Center of superior concha” have the function of “clearing heat.” Zhang et al^[43]^ conducted a meta-analysis on the correlation between the type of Chinese medicine constitution and intestinal polyps, and the results showed that the onset of CPs has a certain connection with the human constitution. Among them, the quality of “Dampness-Heat” is the most common. This result also supports the performance of the functions of the “Kidney,” the “Large Intestine” and the “Center of superior concha” in patients with CPs.

The skin resistance on the surface of the auricle is affected by the anatomical characteristics, the distribution of nerves and blood vessels, the degree of keratinization, and other factors.^[44]^ The cuticle of the auricle is thick, and there are fewer electric ions and no body fluids in the epidermis, so the resistance of the auricle is usually relatively high. In a study conducted by Oleson^[45]^ on patients with musculoskeletal pain, he found that the changes in skin conductivity in the pain area of the body were attributed to the local overactivity of the sympathetic nervous system, which at the same time caused increased activities of skin tissues and accelerated metabolism. This increased various substances, such as sweat glands and sebaceous glands, penetrating the epidermis from the dermis. Since these substances are all electrolytes that can dissociate into positive and negative charges, the skin potential increases and the resistance decreases.^[46]^ The auricular point electro-measuring instrument is designed according to this principle. More and more new technologies are being applied in this field, so that the invention of auricular detection instruments continues to emerge. Huang et al^[47]^ reported a 3D graphene-based ear-conformable sensing device with embedded and distributed 3D electrodes for physiological monitoring of the entire auricle. It demonstrated the spatiotemporal auricle electrical skin resistance (AESR) mapping for the first time, observing the subject-specific AESR distribution in more than 30 cases. This 3D electronic platform and AESR-based biometric findings show certain prospects for biomedical applications.

From the results of this study, it can be seen that there was no difference in the electrical characteristics of the auricular points of the subjects’ left and right ears. It may be due to the close anatomical characteristics of the human ear, that is, the distribution of cartilage, fat, nerves, and blood vessels is very close. In addition, the factors affecting the electrical conductivity of both ears are similar, such as external factors, wind speed, temperature, magnetic field, etc, as well as internal factors, hemorrheology and cuticle.^[44]^

### 5.4. Tenderness testing

Compared with the control group, patients with CPs showed significant tenderness in the “Duodenum,” the “Large Intestine,” the “Small Intestine,” and the “Center of superior concha.” Among them, the “Large Intestine” has a more significant predictive ability for CPs, which is also consistent with the results obtained by electrical measurement. Chinese medicine uses the word “Tong” to indicate that the overall function of the human body is in a state of normal operation. Based on this, there is the theory of “no ‘Tong’ leading Pain,” which essentially means that under the influence of some pathogenic factors, the human body’s qi and blood are not smooth, resulting in stasis, stagnation, and local pain such as zang-fu organs and meridians.^[48]^

CPs are not clearly defined in traditional Chinese medicine theories. But generally speaking, the main pathogenesis of CPs is caused by the condensation or accumulation of evil gas, such as phlegm, stasis, dampness, and poison, on the basis of “Zheng qi” deficiency.^[49]^ This is reflected in the obvious tenderness of the “Duodenum,” the “Large Intestine,” and the “Small Intestine.” In general, the degree of tenderness is correlated with the severity of the disease. Those with mild conditions have less pain and fewer tender points. For those with severe conditions, the pain is severe, and there are more tender points. After recovery from illness, the pain points will decrease or disappear. When the disease metastasizes, pain points shift with it.^[34]^ However, whether there is a correlation between the tenderness level of the subjects and the progression of CP disease remains to be further confirmed.

### 5.5. Implications of the findings

The findings of this study enhance our understanding of the relationship between auricular-specific changes and CP status, as well as the importance of finding simple and effective methods to assist in the diagnosis of CPs. In particular, the combination of Western medicine diagnosis and traditional Chinese medicine diagnosis will be more conducive to our cognition of diseases. As a technology with clinical value, auricular point diagnosis is of great significance in disease prevention, screening, and even early treatment. It is worthy of our application in the diagnosis and treatment of intestinal diseases, such as early diagnosis and long-term follow-up of CPs, and even the screening of patients at high risk of colorectal cancer.

### 5.6. Limitations of this study

This study was a single-center study carried out in a gastroendoscopic center. There were more patients with digestive system diseases, and patients with undiagnosed CPs could not be excluded from other colorectal diseases, which may have a certain impact on the results. Therefore, it is necessary to conduct a multicenter, large-sample study for further clinical validation. At the same time, although this study confirmed that there was a certain correlation between specific changes of the auricular point and CPs, it was difficult to determine the relationship between the emergence time of auricular point signals and the progression and severity of the disease. Based on this, further studies need to be carried out. It is necessary to further discuss the number of polyps, the size of polyps, the lesion location, the pathological type, and the history of polyps in CPs patients. It is also necessary to conduct stratified studies on polyp patients of different age groups.

## 6. Conclusion

Compared with people without CPs, patients with CPs have a greater risk of cancer. Therefore, screening and early diagnosis of CPs are very important. However, the lack of simple and feasible auxiliary diagnostic methods in clinical practice makes the early detection and treatment of CPs difficult. This study showed that the specific changes of some auricular points, such as the “Large Intestine,” the “Rectum,” the “Small Intestine,” the “Center of Superior Concha,” the “Shenmen,” and the “Kidney” were closely related to CPs. Through the above analysis, it is suggested that the above auricular points can be used as the clinical basis for the diagnosis and treatment of CPs, and the clusters of auricular point-specific changes for CP screening can be established. It is even expected to achieve early screening of CPs by establishing a comprehensive analysis model of auricular points and further carrying out clinical validation experiments.

## Author contributions

**Conceptualization:** Longshu Zhang, Ying Chen, Hui Li, Lirong Zhou, Mengyan Zhou, Xixia Zhang.

**Data curation:** Longshu Zhang, Ying Chen, Hui Li, Lirong Zhou, Mengyan Zhou, Xixia Zhang.

**Formal analysis:** Longshu Zhang.

**Funding acquisition:** Xixia Zhang.

**Investigation:** Longshu Zhang, Ying Chen, Hui Li, Lirong Zhou, Mengyan Zhou, Xixia Zhang.

**Methodology:** Longshu Zhang, Hui Li, Lirong Zhou, Xixia Zhang.

**Project administration:** Longshu Zhang.

**Resources:** Longshu Zhang.

**Software:** Longshu Zhang.

**Supervision:** Longshu Zhang, Hui Li, Xixia Zhang.

**Validation:** Longshu Zhang.

**Visualization:** Longshu Zhang.

**Writing – original draft:** Longshu Zhang, Ying Chen, Mengyan Zhou.

**Writing – review & editing:** Longshu Zhang, Mengyan Zhou.
